# Differentiation of renal angiomyolipoma without visible fat from small clear cell renal cell carcinoma by using specific region of interest on contrast-enhanced CT: a new combination of quantitative tools

**DOI:** 10.1186/s40644-021-00417-3

**Published:** 2021-07-05

**Authors:** Xu Wang, Ge Song, Haitao Jiang

**Affiliations:** 1 Department of Radiology, Cancer Hospital of the University of Chinese Academy of Sciences (Zhejiang Cancer Hospital), No 1, Banshan East Road, Hangzhou, Zhejiang Province 310022 People’s Republic of China; 2 Institute of Cancer and Basic Medicine, Chinese Academy of Sciences, No 1, Banshan East Road, Hangzhou, Zhejiang Province 310022 People’s Republic of China

## Abstract

**Background:**

To investigate the value of using specific region of interest (ROI) on contrast-enhanced CT for differentiating renal angiomyolipoma without visible fat (AML.wovf) from small clear cell renal cell carcinoma (ccRCC).

**Methods:**

Four-phase (pre-contrast phase [PCP], corticomedullary phase [CMP], nephrographic phase [NP], and excretory phase [EP]) contrast-enhanced CT images of AML.wovf (n = 31) and ccRCC (n = 74) confirmed by histopathology were retrospectively analyzed. The CT attenuation value of tumor (AVT), net enhancement value (NEV), relative enhancement ratio (RER), heterogeneous degree of tumor (HDT) and standardized heterogeneous ratio (SHR) were obtained by using different ROIs [small: ROI (1), smaller: ROI (2), large: ROI (3)], and the differences of these quantitative data between AML.wovf and ccRCC were statistically analyzed. Multivariate regression was used to screen the main factors for differentiation in each scanning phase, and the prediction models were established and evaluated.

**Results:**

Among the quantitative parameters determined by different ROIs, the degree of enhancement measured by ROI (2) and the enhanced heterogeneity measured by ROI (3) performed better than ROI (1) in distinguishing AML.wovf from ccRCC. The receiver operating characteristic (ROC) curves showed that the area under the curve (AUC) of RER_CMP (2), RER_NP (2) measured by ROI (2) and HDT_CMP and SHR_CMP measured by ROI (3) were higher (AUC = 0.876, 0.849, 0.837 and 0.800). Prediction models that incorporated demographic data, morphological features and quantitative data derived from the enhanced phase were superior to quantitative data derived from the pre-contrast phase in differentiating between AML.wovf and ccRCC. Among them, the model in CMP was the best prediction model with the highest AUC (AUC = 0.986).

**Conclusion:**

The combination of quantitative data obtained by specific ROI in CMP can be used as a simple quantitative tool to distinguish AML.wovf from ccRCC, which has a high diagnostic value after combining demographic data and morphological features.

## Introduction

Renal tumors with a diameter of 4 cm or less are usually called small renal tumors in clinical practice [1]. With the development of imaging technology and the improvement of health awareness, the detection of small renal tumors has been increasing year by year in recent years [2]. As many as 30% of patients over the age of 50 have at least one incidental renal lesion on imaging [3]. Renal tumors are divided into benign renal tumors and malignant renal tumors. The most common malignant renal tumor is clear cell renal cell carcinoma (ccRCC), accounting for about 80% of malignant renal tumors [4], while the most common benign renal tumor is renal angiomyolipoma (AML), accounting for about 44% of benign renal tumors [5]. For most AMLs, visible fat can be detected on CT or MR [6], but for about 5% of AMLs that have less than 10% fat component, the fat is difficult to detect with imaging, and these are called angiomyolipoma without visible fat (AML.wovf) or angiomyolipoma with minimal fat [7–9]. This type of AML is radiologically prone to misdiagnosed as ccRCC which could lead to major treatment and outcome differences. ccRCC may be treated with radical nephrectomy, partial nephrectomy, ablation, or active surveillance, while small AML.wovf may only require regular follow-up. Considering that the proportion of benign tumors in small (≤ 4 cm) renal tumors is higher [10, 11], correct differential diagnosis is very important for the choice of treatment option and to avoid unnecessary surgery.

CT is the first-line imaging method for the diagnosis of renal tumors [11–13]. Analyzing various qualitative and quantitative features on CT can improve the accuracy of differential diagnosis between AML.wovf and ccRCC, which is the most widely used diagnostic method in most medical centers. Since the sensitivity of morphological feature determination is low and subjective to experience [14, 15], many previous studies have used the imaging features of contrast-enhanced CT to distinguish these two tumors, especially the attenuation degree and homogeneity of enhancement [16, 17]. Some studies showed that there are differences in the attenuation degree and homogeneity of enhancement between the two tumors: the attenuation degree of ccRCC is generally higher than that of AML.wovf [18], and ccRCC is mostly heterogeneously enhancing while AML.wovf is more homogeneously enhancing [19]. However, some studies have shown that there is no significant difference in the attenuation degree and homogeneity of enhancement between ccRCC and AML.wovf [20–23]. Because there is no uniform reference standard, these studies use different methods to select the ROI for quantitative measurement, which results in differences among some research results. Moreover, the determination of tumor heterogeneity is mostly based on subjective judgment, so its clinical application is limited.

Rosenkrantz et al. [24] studied the selection method of the ROI in differentiating renal cyst from RCC and found that the size of the ROI would affect the diagnostic efficiency. To the best of our knowledge, there have been no studies to distinguish ccRCC and AML.wovf based on different ROIs from contrast-enhanced CT. This study will investigate the value of quantitative parameters determined by different ROIs in differentiating ccRCC and AML.wovf for the first time, with the aim of providing a simple quantitative tool for daily routine differential diagnosis.

## Materials and methods

### Patient cohort

This study was a retrospective case–control study. It was approved by Ethical Committee of our Hospital, and the requirement for written informed consent was waived because of the retrospective nature of the study. The images used in this study were all anonymized.

Between October 2016 and April 2020, 358 patients with suspected renal tumors underwent CT examination before surgery. The inclusion criteria of this study were as follows: (1) The maximum cross-sectional diameter of the tumor on CT image should be 4 cm or less; (2) The patients underwent four-phase (pre-contrast phase [PCP], corticomedullary phase [CMP], nephrographic phase [NP], and excretory phase [EP]) CT scan in our department within 2 weeks before the operation; (3) The CT slice thickness was 1 mm or less; (4) There was no obvious visual macroscopic fat within the renal masses on CT (PCP); (5) Surgical histopathology confirmed either AML or ccRCC in all lesions.

253 patients were excluded because: (1) The maximum diameter of the tumor was more than 4 cm (n = 104); (2) Incomplete four-phase CT (n = 27) of which 12 patients with missing NP and 15 patients with missing EP; (3) The CT slice thickness was more than 1 mm (n = 68); (4) Tumors had typical macroscopic fat on CT (PCP) (n = 16); (5) Histopathological diagnosis of lesions was neither AML nor ccRCC (n = 38), including papillary RCC (n = 15), chromophobe RCC (n = 12), urothelial cancer (n = 4), metastasis (n = 1), and oncocytoma (n = 6).

**Fig. 1:**
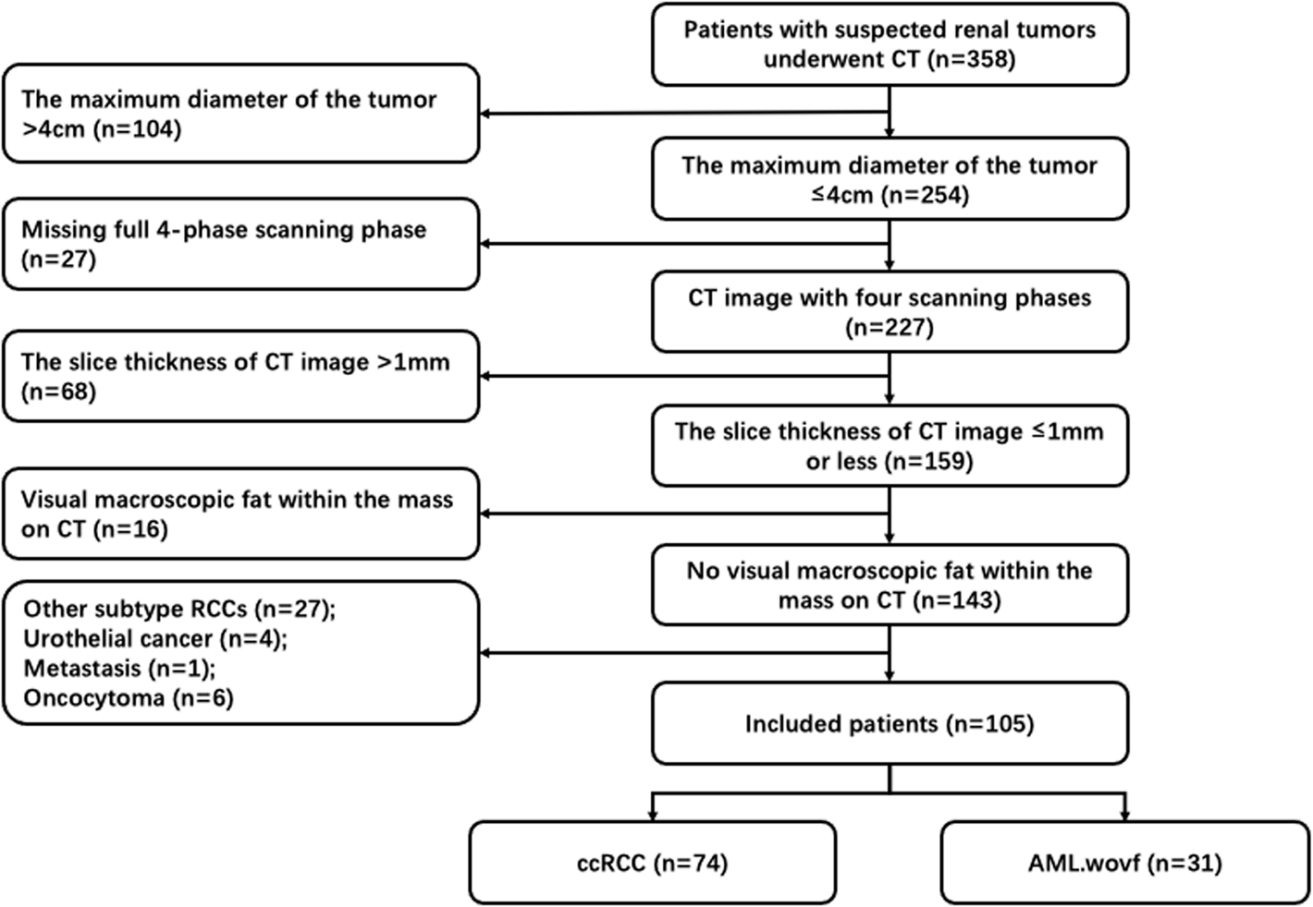
Flowchart of patient inclusion and exclusion

As a result, our cohort comprised of 105 patients (mean age, 53.55 years; age range, 13–81 years) with 105 small renal tumors. They were divided into ccRCC group (n = 74) and AML.wovf group (n = 31) (Fig. 1).

### CT examination

All patients were examined by a spiral CT scanner with 64-row detectors (Siemens Somatom Definition Flash, Siemens AG). Automated tube current modulation based on the patient’s body weight was used, and the CT scanning parameters were follows: the tube voltage was 120 kV, the collimation width was 0.625 mm, the scanning thickness was 5 mm, and the reconstruction thickness was 1 mm. The PCP, CMP, NP, and EP of the CT examination were acquired for each patient with the following protocol: the PCP was performed first, and then a nonionic contrast agent (Ultravist 370, Bayer Schering Pharma AG) was injected intravenously utilizing a high-pressure syringe at a rate of 4.5 ml/s for enhanced scan. CMP, NP and EP were obtained post contrast administration by applying bolus tracking (CMP: 7 s after the attenuation value of aorta reached 100 Hu, NP: 30 s after CMP, EP: 60–180 s after NP).

### Analysis of morphological features

Blinded consensus reading was performed by two radiologists with 10 and 12 years of experience in abdominal imaging. Image analysis was performed utilizing a radiology information system/picture archiving and communication systems (RIS/PACS, Greenlander, Mindray Health) and by assessing axial and multi-planar reconstructed (MPR) images. The analysis content included location (left kidney/right kidney), growth pattern of tumor (endophytic/exophytic), pseudocapsule sign, cystic degeneration and angular interface. The pseudocapsule sign, cystic degeneration and angular interface were determined in NP or EP. The pseudocapsule sign on CT imaging was defined as the low attenuation rim around the mass which likely correlates to histopathologically proven fibrous tissue surrounding the renal mass. [25]. The angular interface was defined as the pyramidal interface between the parenchymal portion of a mass and the surrounding tissue, and with an angle of 90° or less [14, 25].

### Determination of quantitative parameters

**Fig. 2:**
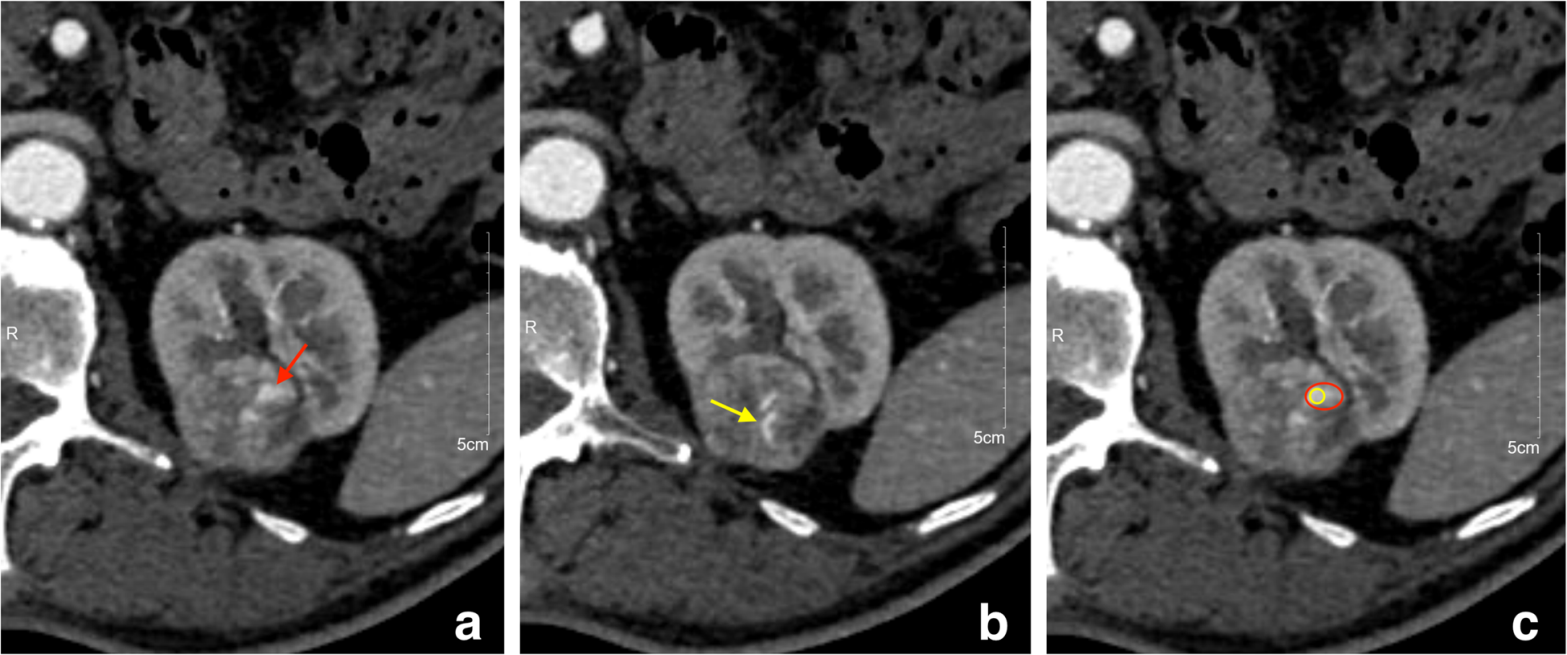
The placement method of ROI (1) and ROI (2). **a** and **b** are schematic diagram of blood-rich supply region in the tumor: significantly enhanced region was observed as small patches with unclear boundary at the edge of tumor's parenchyma (red arrow), while tumor vessels presented an arc-shaped linear structure (yellow arrow). (c) is definition method of ROI (1) and ROI (2) based on (a): red ROI is ROI (1), and yellow ROI is ROI (2)

In order to decrease the difference between observers, two radiologists placed region of interest (ROI) on the axial image with 1 mm slice thickness to determine the quantitative parameters of the tumor. Firstly, two different ROIs [ROI (1) and ROI (2)] were used to determine the attenuation value of tumor (AVT) in CMP, and the attenuation value of cortex (AVC) was measured at the adjacent renal cortex where the enhancement was homogeneous. Both ROI (1) and ROI (2) were placed on the portion of the tumor demonstrating the greatest enhancement, with an area of about 50 ~ 100 mm^2^ for ROI (1) and 10 ~ 20 mm^2^ for ROI (2) (Fig. 2). The selection principles of ROI (1) & ROI (2) are as follows: (1) the selection of ROI in PCP, NP and EP should refer to CMP and place similar-sized ROI at similar level as far as possible; (2) avoid intratumor calcification, cystic degeneration and vessels as far as possible; (3) if there are multiple regions with obvious enhancement in the lesion, measure the value of each enhanced region respectively, and then take their average value; (4) select three adjacent section-images, each section-image was measured at least twice, and take the average value. Finally, the average value independently measured by two radiologists was used as AVT. The net enhancement value (NEV) and relative enhancement ratio (RER) of tumor were calculated respectively as follows:

$${\text{NEV}} = {\text{AVT}}\_{\text{CMP}}/{\text{NP}}/{\text{EP}}{-}{\text{ AVT}}\_{\text{PCP}}$$

$${\text{RER}} = {\text{AVT}}\_{\text{CMP}}/{\text{NP}}/{\text{EP}} / {\text{AVC}}\_{\text{CMP}}/{\text{NP}}/{\text{EP}} \times {\text{1}}00$$

**Fig. 3:**
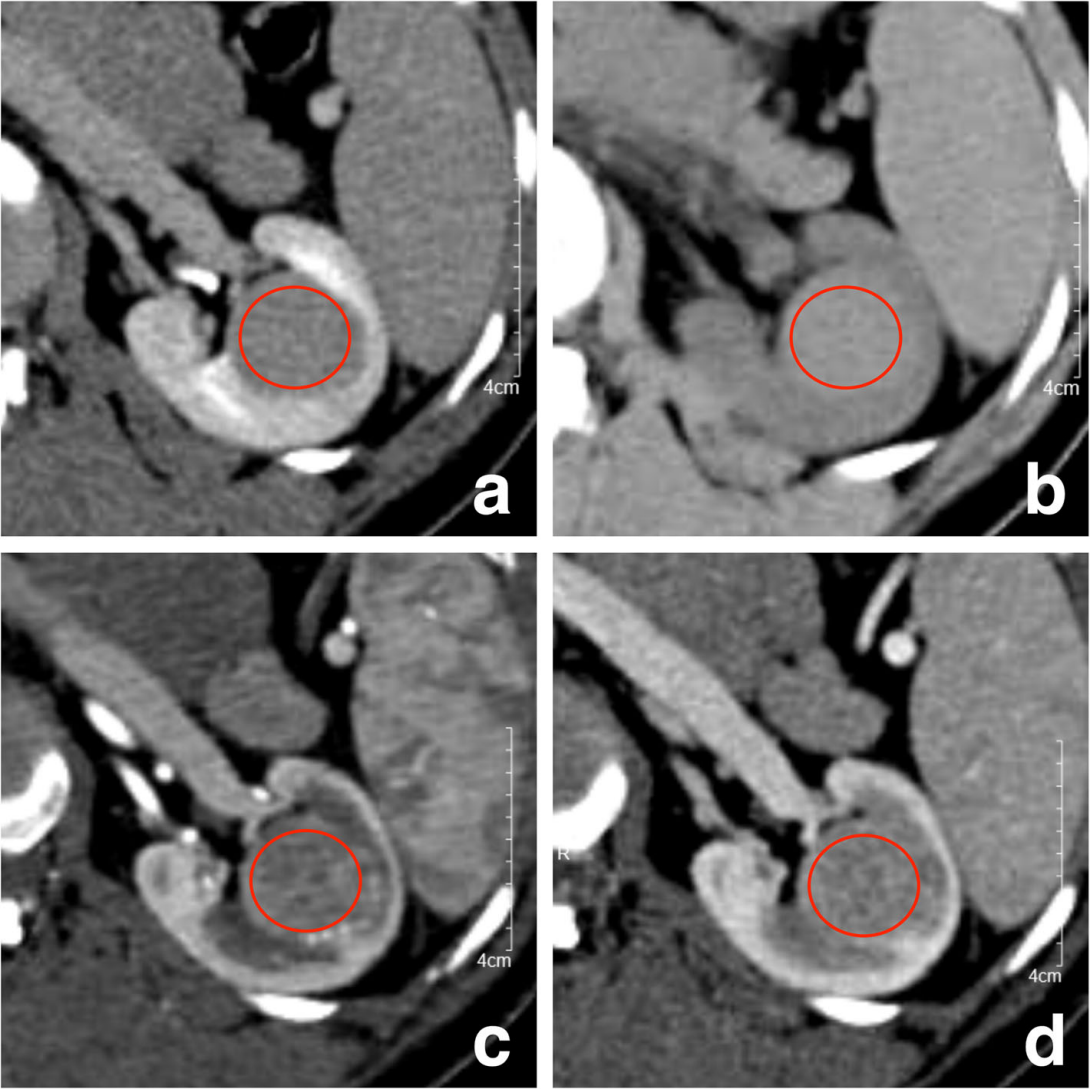
The placement method of ROI (3). **a**–**d** are EP, PCP, CMP and NP images, respectively: firstly, the tumor boundary should be determined in EP, and ROI (red) should cover the entire range of tumor as much as possible, with its edge 2–3 mm away from the tumor's edge; secondly, ROI of the same size should be placed in PCP, CMP and NP according to the location of ROI in EP

ROI (3) was placed on the largest cross-sectional image of the tumor to measure the heterogeneous degree (HD), which was recorded as standard deviation (SD) of the CT attenuation value measured by ROI (3). Since the boundary of tumor were most clearly shown in EP, the HD of tumor (HDT) was measured at this phase first, and the HD of psoas (HDP) was measured at the same level. The determination of HDT in PCP, CMP and NP should refer to the position of ROI in EP (Fig. 3). The selection principles of ROI (3) are as follows: (1) the shape of ROI was circular or quasi-circular; (2) ROI should cover the whole range of tumor as much as possible (included all components of the tumor), and its edge should be 2–3 mm away from the edge of tumor, avoiding be placed outside of the tumor; (3) each section-image was measured three times and take their average value. The standardized heterogeneous ratio (SHR) of tumor in each phase was calculated respectively, which was defined as HDT/ HDP × 100. The AVT, NEV and RER of each phase measured by ROI (3) were also recorded.

### Statistical analysis

All data analysis was performed using IBM SPSS Statistics (version 26.0, SPSS Inc.). Chi-square test was used to compare the qualitative data. Interclass correlation coefficient (ICC) was used to evaluate the inter-observer agreement of quantitative parameters. Firstly, Kolmogorov–Smirnov test was used to evaluate the normality of quantitative data. The independent sample *t* test was used for the comparison of quantitative data conforming to the normal distribution, while Mann–Whitney U test was used for the comparison of quantitative data which did not conform to normal distribution. The receiver operating characteristic (ROC) curves were constructed and the area under the curve (AUC) was calculated to evaluate the diagnostic efficacy of statistically significant quantitative parameters. Multivariate analysis was performed on demographic data, morphological features, and quantitative parameters with significant difference by using logistic regression. The main factors were screened for differentiating AML.wovf and ccRCC in each scanning phase, and prediction models were established based on four scanning phases. The sensitivity, specificity and accuracy of the model were calculated, while the AUC and its 95% confidence interval of ROC curve were also calculated to evaluate predictive performance of the model. *P* < 0.05 were considered statistically significant.

## Results

### Demographic data and morphological features

There were 74 patients with ccRCCs (48 males and 26 females; mean age, 54.61 years; age range, 29–81 years) and 31 patients with AML.wovfs (7 males and 24 females; mean age, 51.00 years; age range, 13–71 years) in our cohort. There was significant statistical difference on gender between the two tumors (*P* < 0.01). AML.wovf was significantly more common in females, while ccRCC was more common in males. There was no significant difference in age between the two tumors(*P* > 0.05)(Table 1).

**Table 1:** The demographic data and morphological features of ccRCC and AML.wovf

	ccRCC (n = 74)	AML.wovf (n = 31)	χ^2^/*t*	*P*
Age (years)	54.61 ± 11.44	51.00 ± 10.71	1.508*	0.135
Size (cm)	2.80 ± 0.71	2.70 ± 0.62	0.666*	0.507
Gender			15.661	< 0.001
Male	48 (64.9)	7 (22.6)		
Female	26 (35.1)	24 (77.4)		
Location			0.205	0.651
Left kidney	37 (50.0)	17 (54.8)		
Right kidney	37 (50.0)	14 (45.2)		
Growth pattern of tumor			2.849	0.091
Endophytic	42 (56.8)	12 (38.7)		
Exophytic	32 (43.2)	19 (61.3)		
Pseudocapsule sign			8.544	0.003
Observed	25 (33.8)	2 (6.5)		
Not observed	49 (66.2)	29 (93.5)		
Cystic degeneration			7.812	0.005
Observed	36 (48.6)	6 (19.4)		
Not observed	38 (51.4)	25 (80.6)		
Angular interface			11.693	0.001
Observed	9 (12.2)	13 (41.9)		
Not observed	65 (87.8)	18 (58.1)		

ccRCC: clear cell renal cell carcinoma, AML.wovf: angiomyolipoma without visible fat

*t value

Data are numbers of patients with a given tumor. Data in parentheses are percentages. Age and diameter are means ± standard deviations

The independent sample *t*-test was applied in the analysis of age and diameter comparisons, the chi-square test was applied in the rest of the comparisons

**Fig. 4:**
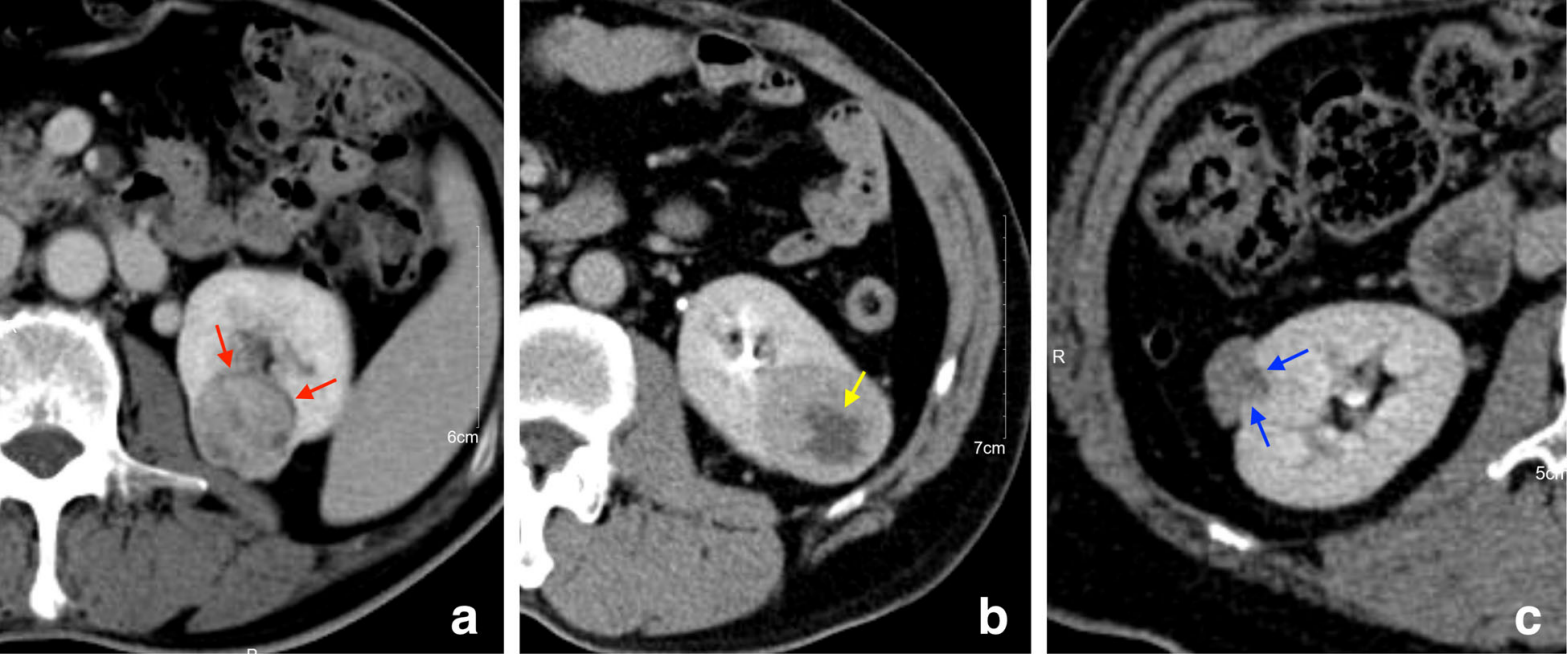
Schematic diagram of the morphological features of ccRCC and AML.wovf. **a**, a 70-year-old male with histopathologically proven ccRCC. Pseudocapsule sign is shown as a low attenuation ring at the edge of tumor (red arrow). **b**, a 57-year-old male with histopathologically proven ccRCC. Cystic degeneration is observed in the central area of tumor (yellow arrow). **c**, a 49-year-old female with histopathologically proven AML.wovf. Angular interface with an angle less than 90° is observed between the tumor and the surrounding tissue (blue arrow)

The psuedocapsule sign (25 out of 74 versus 2 out of 31) and cystic degeneration (36 out of 74 versus 6 out of 31) were seen more frequently in ccRCCs compared to AML.wovfs. In contrary, an angular interface was less commonly seen in ccRCCs compared to AML.wovfs (9 out of 74 versus 13 out of 31) (Fig. 4). The difference of these morphological features was statistically significant (*P* < 0.05). There was no statistical difference in size, location, growth pattern between the two tumors (*P* > 0.05) (Table 1).

### The inter-observer agreement

We compared the quantitative data measured by two radiologists, and the ICC was found to be 0.808 to 0.961. Among them, the ICC of AVT_CMP, AVT_NP and AVT_EP measured by ROI (1) were 0.921, 0.886 and 0.852 (*P* < 0.001), the ICC of AVT_CMP, AVT_NP and AVT_EP measured by ROI (2) were 0.881, 0.874 and 0.827 (*P* < 0.001), respectively. An ICC greater than 0.75 was considered to be of good agreement.

### Quantitative data

AVT_PCP (1), AVT_CMP (1), NEV_CMP (1), RER_CMP (1), and RER_NP (1) determined by ROI (1) showed statistical differences between ccRCC and AML.wovf (*P* < 0.05). There was no statistical difference between AVT_NP (1), NEV_NP (1) and quantitative parameters of EP determined by ROI (1) (*P* > 0.05). Among the quantitative parameters determined by ROI (2), only AVT_EP (2) had no statistical difference (*P* > 0.05), and the remaining quantitative parameters of ccRCC were all statistically higher than AML.wovf (*P* < 0.05). In terms of heterogeneous degree, HDT and SHR of ccRCC determined by ROI (3) were statistically higher than AML.wovf in each enhanced phase (*P* < 0.05). There was no statistically significant difference between the two tumors in HDT_PCP, SHR_PCP, and enhancement degree of each phase determined by ROI (3) (*P* > 0.05) (Table 2).

**Table 2:** The comparative analysis on quantitative parameter of ccRCC and AML.wovf

Parameter	ccRCC (n = 57)	AML.wovf (n = 28)	*t*/*Z*	*P*
*Pre-contrast phase*				
AVT_PCP (1)	32.78 ± 6.50	38.33 ± 5.47	− 4.171	< 0.001
AVT_PCP (2)	34.73 ± 6.08	40.17 ± 6.43	− 4.115	< 0.001
AVT_PCP (3)	30.28 ± 5.78	32.72 ± 9.18	− 1.371	0.178
HDT_PCP	11.60 ± 2.42	12.78 ± 4.91	− 1.269	0.213
SHR_PCP	150.12 ± 38.98	166.87 ± 60.86	− 1.416	0.165
*Corticomedullary phase*				
AVT_CMP (1)	166.15 ± 40.61	147.58 ± 27.16	2.735	0.008
NEV_CMP (1)	133.37 ± 40.30	108.94 ± 28.39	3.529	0.001
RER_CMP (1)	88.22 ± 19.03	76.23 ± 14.59	3.496	0.001
AVT_CMP (2)	216.91 ± 43.87	181.18 ± 32.67	4.596	< 0.001
NEV_CMP (2)	182.31 ± 45.15	140.68 ± 33.75	5.191	< 0.001
RER_CMP (2)	116.21 ± 17.48	89.39 ± 14.48	7.522	< 0.001
AVT_CMP (3)	127.99 ± 41.55	115.54 ± 31.79	1.494	0.138
NEV_CMP (3)	95.47 ± 34.12	85.40 ± 29.43	1.524*	0.127
RER_CMP (3)	66.85 ± 19.44	60.75 ± 14.96	1.562	0.121
HDT_CMP	39.58 ± 8.38	29.04 ± 6.92	6.174	< 0.001
SHR_CMP	412.11 ± 116.92	290.53 ± 85.78	5.224	< 0.001
*Nephrographic phase*				
AVT_NP (1)	108.05 ± 22.28	108.77 ± 21.26	− 0.039*	0.969
NEV_NP (1)	75.27 ± 22.51	70.13 ± 21.61	1.081	0.282
RER_NP (1)	72.84 ± 12.38	67.16 ± 7.94	2.802	0.006
AVT_NP (2)	129.11 ± 24.69	116.90 ± 21.80	2.390	0.019
NEV_NP (2)	94.25 ± 25.98	78.02 ± 26.48	2.903	0.005
RER_NP (2)	87.36 ± 12.75	71.83 ± 8.49	7.307	< 0.001
AVT_NP (3)	92.89 ± 27.15	93.20 ± 23.71	− 0.054	0.957
NEV_NP (3)	62.67 ± 25.87	60.48 ± 21.26	0.941*	0.347
RER_NP (3)	62.52 ± 17.48	57.28 ± 10.16	1.917	0.058
HDT_NP	25.03 ± 5.86	20.19 ± 5.21	3.977	< 0.001
SHR_NP	304.39 ± 80.20	259.94 ± 64.79	2.329*	0.020
*Excretory phase*				
AVT_EP (1)	87.56 ± 16.61	87.72 ± 12.03	− 0.051	0.960
NEV_EP (1)	54.77 ± 17.10	49.07 ± 13.18	1.660	0.100
RER_EP (1)	63.73 ± 9.27	60.52 ± 10.64	1.550	0.124
AVT_EP (2)	101.46 ± 18.53	97.05 ± 14.63	1.178	0.242
NEV_EP (2)	66.86 ± 19.54	56.56 ± 18.08	2.518	0.013
RER_EP (2)	73.95 ± 10.30	65.74 ± 11.32	3.616	< 0.001
AVT_EP (3)	80.49 ± 17.99	78.64 ± 19.51	0.468	0.641
NEV_EP (3)	50.67 ± 17.70	45.92 ± 16.21	1.637*	0.102
RER_EP (3)	57.88 ± 11.35	53.85 ± 11.32	1.663	0.099
HDT_EP	19.79 ± 4.72	16.00 ± 4.40	3.833	< 0.001
SHR_EP	247.34 ± 66.58	204.87 ± 54.68	3.134	0.002

ccRCC: clear cell renal cell carcinoma, AML.wovf: angiomyolipoma without visible fat

PCP: pre-contrast phase, CMP: corticomedullary phase, NP: nephrographic phase, EP: excretory phase

AVT: attenuation value of tumor, NEV: net enhancement value, RER: relative enhancement ratio, HDT: heterogeneous degree of tumor, SHR: standardized heterogeneous ratio

(1): ROI (1), (2): ROI (2), (3): ROI (3)

*: *Z* value

Data of AVT, HDT and NEV are means ± standard deviations in Hounsfield units. Data of RER and SHR are means ± standard deviations in percentage ratio

The Mann–Whitney U test was applied in the analysis of AVT_NP (1), NEV_CMP (3), NEV_NP (3), SHR_NP and NEV_EP (3) comparison, the independent sample *t*-test was applied in the rest of the comparisons

ROC curves showed that the AUC of RER_CMP (2), RER_NP (2), HDT_CMP and SHR_CMP were higher (0.876, 0.849, 0.837 and 0.800, respectively), and the AUC of other quantitative parameters were all lower than 0.80 (Table 3). In terms of enhanced attenuation value, the quantitative parameters determined by ROI (2) have better diagnostic performance than ROI (1) and ROI (3).

**Table 3:** Results of receiver operating characteristic (ROC) curves for quantitative data

Parameter	AUC	95% CI	
		Lower bound	Upper bound
AVT_PCP (1)	0.758	0.659	0.858
AVT_CMP (1)	0.644	0.535	0.752
NEV_CMP (1)	0.684	0.579	0.789
RER_CMP (1)	0.697	0.596	0.799
RER_NP (1)	0.638	0.531	0.744
AVT_PCP (2)	0.744	0.635	0.854
AVT_CMP (2)	0.739	0.641	0.836
NEV_CMP (2)	0.760	0.667	0.853
RER_CMP (2)	0.876	0.809	0.942
AVT_NP (2)	0.639	0.511	0.767
NEV_NP (2)	0.665	0.535	0.795
RER_NP (2)	0.849	0.773	0.924
NEV_EP (2)	0.634	0.512	0.756
RER_EP (2)	0.703	0.585	0.820
HDT_CMP	0.837	0.758	0.915
SHR_CMP	0.800	0.710	0.891
HDT_NP	0.721	0.611	0.831
SHR_NP	0.645	0.532	0.758
HDT_EP	0.726	0.616	0.835
SHR_EP	0.688	0.577	0.798

AUC: area under curve, CI: confidence interval

PCP: pre-contrast phase, CMP: corticomedullary phase, NP: nephrographic phase, EP: excretory phase

AVT: attenuation value of tumor, NEV: net enhancement value, RER: relative enhancement ratio, HDT: heterogeneous degree of tumor, SHR: standardized heterogeneous ratio

(1): ROI (1), (2): ROI (2)

### Multivariate analysis and prediction models

Demographic data, morphological signs, and quantitative parameters with *P* < 0.05 were used for multivariate analysis by using logistic regression. The results showed that gender, pseudocapsule sign, angular interface and AVT_PCP (1) were the main factors for differentiating AML.wovf and ccRCC in PCP (*P* < 0.05); gender, cystic degeneration, RER_CMP (2) and SHR_CMP were the main factors for differentiating two tumors in CMP (*P* < 0.05); gender, pseudocapsule sign, RER_NP (2) and HDT_NP were the main factors for differentiating two tumors in NP (*P* < 0.05); gender, angular interface, RER_EP (2) and SHR_EP were the main factors for differentiating two tumors in EP (*P* < 0.01) (Table 4).

**Table 4:** Multivariate regression analysis of pre-contrast and contrast-enhanced phase

Model	Coefficient	Odds ratio	95% CI (odds ratio)		*P*
			Lower bound	Upper bound	
*Pre-contrast phase*					
Constant	5.257				
Gender	3.137	23.028	4.892	108.408	0.000
Pseudocapsule sign	2.549	12.801	1.453	112.806	0.022
Angular interface	2.155	8.628	1.956	38.059	0.004
AVT_PCP (1)	− 0.215	0.806	0.720	0.903	0.000
*Corticomedullary phase*					
Constant	− 35.318				
Gender	6.796	894.054	11.272	70,915.930	0.002
Cystic degeneration	3.361	28.816	1.803	460.581	0.017
RER_CMP (2)	0.185	1.203	1.075	1.347	0.001
SHR_CMP	0.038	1.039	1.011	1.066	0.005
*Nephrographic phase*					
Constant	− 17.114				
Gender	2.203	7.403	2.326	35.233	0.001
Pseudocapsule sign	2.038	11.703	0.981	75.477	0.048
RER_NP (2)	0.157	1.177	1.081	1.266	0.000
HDT_NP	0.195	1.345	1.052	1.405	0.008
*Excretory phase*					
Constant	− 12.019				
Gender	2.773	16.005	4.033	65.523	0.000
Angular interface	2.059	7.840	1.883	32.643	0.005
RER_EP (2)	0.088	1.092	1.027	1.162	0.005
SHR_EP	1.788	5.978	2.066	17.292	0.001

PCP: pre-contrast phase, CMP: corticomedullary phase, NP: nephrographic phase, EP: excretory phase

AVT: attenuation value of tumor, RER: relative enhancement ratio, HDT: heterogeneous degree of tumor, SHR: standardized heterogeneous ratio

(1): ROI (1), (2): ROI (2)

**Fig. 5:**
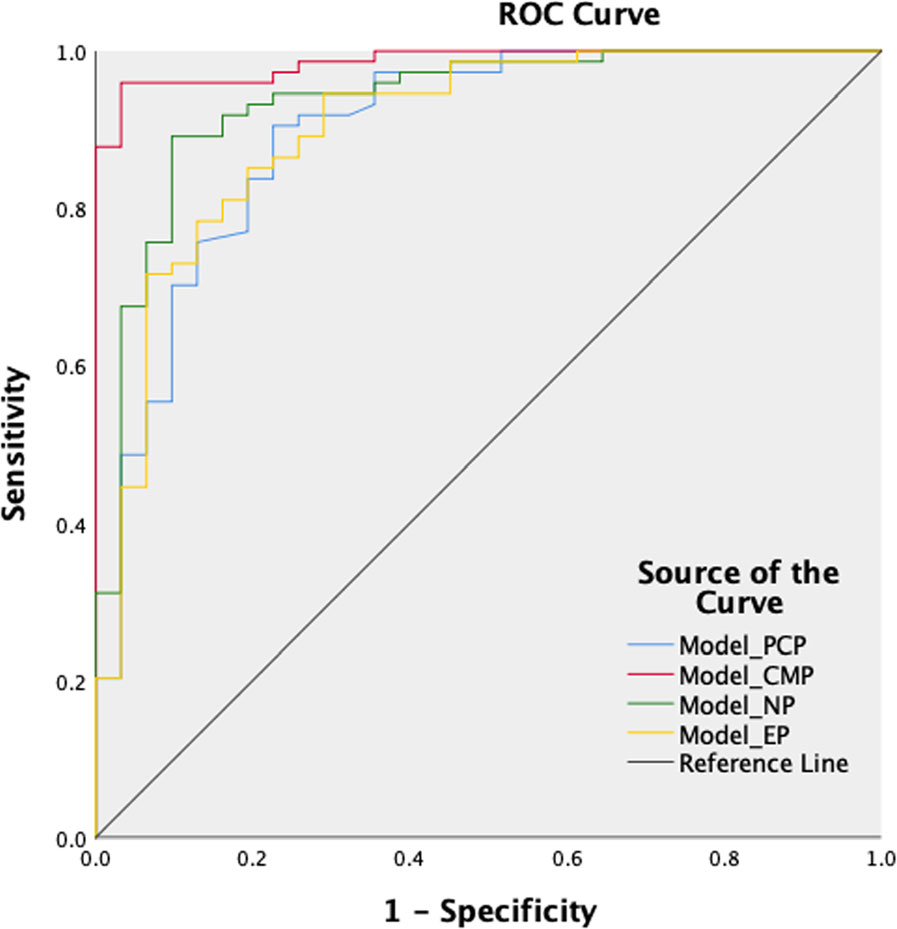
Receiver operating characteristic curves for the prediction models based on four scanning phases

**Fig. 6:**
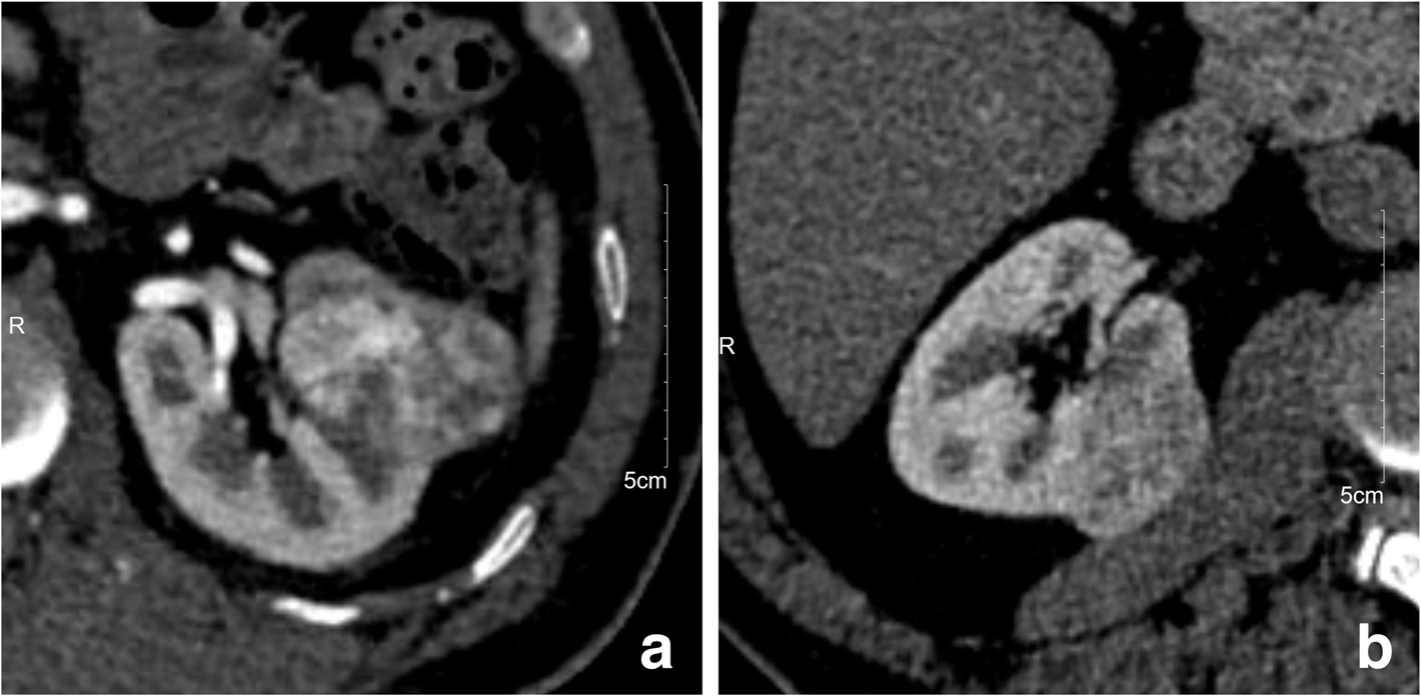
Using CMP images to distinguish AML.wovf from ccRCC. **a**, CMP imaging of a 59-year-old male with histopathologically proven ccRCC. Main quantitative parameter: AVT_CMP (1) = 188.93 Hu, RER_CMP (1) = 92.38, AVT_CMP (2) = 236.74 Hu, RER_CMP (2) = 115.76, HDT_CMP = 43.62 Hu, SHR_CMP = 369.35. **b** CMP imaging of a 55-year-old male with histopathologically proven AML.wovf. Main quantitative parameter: AVT_CMP (1) = 157.75 Hu, RER_CMP (1) = 84.22, AVT_CMP (2) = 179.96 Hu, RER_CMP (2) = 96.08, HDT_CMP = 24.29 Hu, SHR_CMP = 223.46. Generally, it is difficult to distinguish AML.wovf from ccRCC by using only one conventional ROI [ROI (1)] in CMP. However, combining a smaller ROI [ROI (2)] representing the enhancement degree and a larger ROI [ROI (3)] representing the degree of enhanced heterogeneity together can accurately distinguish them in single CMP

The prediction model including demographic data, morphological features and quantitative data showed that the accuracy, sensitivity and specificity of Model_PCP was 84.8%, 93.2% and 64.5% respectively. The accuracy, sensitivity and specificity of Model_CMP were 93.3%, 95.9% and 87.1% respectively. The accuracy, sensitivity and specificity of Model_NP were 89.5%, 94.6% and 77.4% respectively. The accuracy, sensitivity and specificity of Model_EP were 84.8%, 90.5% and 71.0% respectively. The AUC of these four models were 0.898, 0.986, 0.935 and 0.902, respectively (Table 5, Fig. 5). The models in enhanced phase were superior to pre-contrast phase, especially the model in CMP was the best prediction model with excellent diagnostic performance (Fig. 6). In addition, combining the two quantitative parameters after screening can obtain a better differential ability than a single quantitative parameter.

**Table 5:** Diagnostic performance of predictive models based on each scanning phase for differentiating ccRCC and AML.wovf

Model parameter	Model_PCP	Model_CMP	Model_NP	Model_EP
Sensitivity	0.932 (69/74)	0.959 (71/74)	0.946 (70/74)	0.905 (67/74)
Specificity	0.645 (20/31)	0.871 (27/31)	0.774 (24/31)	0.710 (22/31)
Positive predictive value	0.863 (69/80)	0.960 (72/75)	0.909 (70/77)	0.882 (67/76)
Negative predictive value	0.800 (20/25)	0.900 (27/30)	0.857 (24/28)	0.759 (22/29)
Accuracy	0.848 (89/105)	0.933 (98/105)	0.895 (94/105)	0.848 (89/105)
AUC	0.898	0.986	0.935	0.902
AUC (95% CI)				
Lower bound	0.828	0.970	0.881	0.834
Upper bound	0.968	1.000	0.989	0.970

ccRCC: clear cell renal cell carcinoma, AML.wovf: angiomyolipoma without visible fat

AUC: area under curve, CI: confidence interval

Model_PCP is a combined model of gender, pseudocapsule sign, angular interface and AVT_PCP (1)

Model_CMP is a combined model of gender, cystic degeneration, RER_CMP (2) and SHR_CMP. Model_NP is a combined model of gender, pseudocapsule sign, RER_NP (2) and HDT_NP

Model_EP is a combined model of gender, angular interface, NEV_EP (2) and HDT_EP

Values are ratios of the numerator and denominator in parentheses

## Discussion

### Enhancement degree

Several studies have shown that quantitative analysis of CT data was helpful to differentiate AML.wovf from ccRCC. Quantitative data, including CT attenuation value, enhancement ratio, wash-in/wash-out ratio and other quantitative parameters, can directly or indirectly reflect the difference of enhancement characteristics between the two tumors, and all these objective quantitative data were obtained through a ROI. Due to the different ROI placement methods, the results of some previous studies were inconsistent. Kim et al. [18] used a ROI with an area of 0.5 ~ 1 cm^2^ and showed that there were differences in CT attenuation value of CMP, percentage enhancement ratio, enhancement change and absolute washout ratio between ccRCC and AML.wovf, but there were no differences in CT attenuation value at early excretory phase. Sung et al. [11] also applied a ROI with 0.5 ~ 1 cm^2^ to measure attenuation value for obtaining the enhancement mode. Xie et al. [26] applied a ROI accounting for about 50–80% of the tumor area, and the result showed that the pre-enhancement value, net enhancement value and enhancement ratio of the two tumors were statistically different. The results of Yang et al. [23] by using a ROI with an area of about 20 mm^2^ showed that there was no statistical difference between ccRCC and AML.wovf in CMP, while there was statistical difference in NP. Takahashi et al. [21] used a ROI with the largest area as possible, and the results showed that there was no significant difference between ccRCC and AML.wovf. Neither Yan et al. [27] nor Ma et al. [22] described the size of ROI, but their results showed no significant difference between the enhancement value of the two tumors in CMP and NP. The difference between these studies indicated that ROIs with different size will affect the role of quantitative parameters in differential diagnosis, which also lead to the lack of clear reference criteria for daily routine diagnosis.

In our study, ROI (1) and ROI (3) were used to simulate previous studies and a smaller ROI (2) was added for comparison. The results showed that the quantitative data determined by ROI (2) with the smallest region had the best diagnostic performance whose value was generally higher than ROI (1) and ROI (3), especially in ccRCC and CMP. In CMP, the blood supply of ccRCC was unbalanced and cystic degeneration rate was high. As the range of ROI (3) included ischemic and cystic degeneration region of tumor, resulting in the lowest enhanced value and the least significant difference between ccRCC and AML.wovf determined by ROI (3). Although the region of ROI (1) was relatively small, it still inevitably included a part of area with poor blood supply or a little distance from the blood supply vessels, even a little cystic degeneration area. Therefore, its diagnostic performance was not as good as ROI (2), which only measured rich blood supply region. Due to the malignant biology of ccRCC, the basement membrane of tumor blood vessels was incomplete and highly permeable, so the contrast agent was more easily and quickly diffused into the adjacent tissue space than in AML.wovf, which making the enhancement range of this region more obvious [26]. By measuring these rich blood supply regions individually, it could reflect their biological characteristics better and maximize the difference in enhancement degree between ccRCC and AML.wovf.

The difficulty of placing ROI (2) is to identify the rich blood supply region. We found that on thin-section image, the rich blood supply regions of tumors tended to be randomly distributed as irregular small pieces or nodules, which were generally located at the edge of the tumors’ obvious enhancement areas, while the tumor vessels presented as thin strips or earthworm-like linear structures with clear boundaries. These two rich blood supply structures can be distinguished by careful identification. Although the placement of ROI (2) has certain requirements for diagnostic experience of radiologists, it can be better recognized by understanding the morphological characteristics of the rich blood supply region combined with 1 mm thin-section image. In our study, there was a good inter-observer agreement between the quantitative data of two radiologists, which reflected good practicability of this placement method. We also found that using ROI (2) with the smallest region in PCP did not improve ability of differentiation, which was similar to Davenport et al. [28].

### Heterogeneous degree

Enhanced homogeneity is another commonly used indicator to distinguish AML.wovf from ccRCC. ccRCC is mostly characterized by heterogeneous enhancement, while AML.wovf is characterized by homogeneous enhancement [15, 16]. However, previous studies on enhanced homogeneity were mostly based on the subjective judgment of radiologists and lacked quantitative indicators, so some results were inconsistent [20–22]. SD is a measure of the dispersion degree of a single data relative to the average value in data set, while SD of CT value reflects the dispersion degree of each pixel value in ROI. A larger SD indicates the greater difference between most pixel values and the average in ROI, which means a higher heterogeneity. Since the SD value of ROI can be obtained directly from most diagnostic workstations, using SD value to quantify heterogeneity of tumor is a simple and convenient method. Jung et al. [29] used SD value to identify different pathological types of RCC and found that SD value of ccRCC was statistically different from other types of RCC.

Our study indicated that HDT and SHR of ccRCC in each enhanced phase were all statistically higher than those of AML.wovf, among which the diagnostic performance of CMP was the best. In CMP, the relatively high cystic degeneration rate and local ischemic change in ccRCC lead to the decrease of pixel value in this region, which was significantly different from significantly enhanced tumor vessels. Moreover, its malignant biological characteristics made the distribution of tumor’s blood supply unbalanced, and the diffusion degree of contrast agent in each region varies greatly, so its SD value was significantly higher than AML.wovf. In NP and EP, the HDT and SHR of ccRCC were also higher than those of AML.wovf, but the difference was lower than that in CMP, which may be related to gradual increase of pixel value and decrease of SD value caused by slow infiltration of contrast agent in partial ischemic region of ccRCC. However, due to the continuous non-enhancing of complete necrosis and cystic degeneration region, the enhanced homogeneity of ccRCC was still different from AML.wovf which was mainly enhanced by smooth muscle. We applied HDP to standardize HDT in our study to reduce the effect of Image noise which could affect the value of SD. Leng et al. [30] showed that although the denoised image would reduce the value of SD and other heterogeneity parameters, it had no effect on identification performance between AML.wovf and ccRCC.

In recent years, texture analysis has been used to quantify the heterogeneity of tumor and to distinguish AML.wovf from ccRCC with the AUC ranging from 0.68 to 0.988, which showed a good classification efficiency and potential [22, 31–34]. Texture features include energy, entropy, uniformity and other indicators to describe homogeneity, which are more comprehensive than the simple SD value, and may be a better quantitative tool to reflect the heterogeneity of tumor. However, the extraction of texture features is relatively complex and cannot be completed on the diagnostic workstation. Meanwhile, texture features extracted from different studies vary greatly, so texture analysis cannot be widely used in daily routine diagnosis at present. Most of the previous texture analysis showed that the difference between AML.wovf and ccRCC was most significant in PCP, not in enhanced scanning phase. But in fact, enhanced scanning phase may be the best phase to reflect the difference between these two tumors, because the attenuation value of them in PCP overlap partially [21, 35], part of ccRCC can show high attenuation and homogeneity in PCP [20, 26], while some AML.wovf can also show equal attenuation in PCP and most of AML.wovf were detected in enhanced CT [7, 8, 15]. Moreover, texture analysis of whole tumor cannot focus on the single region with most significant enhancement in enhanced scanning phase, which may be the region that can best reflect the difference between these two tumors, because the local heterogeneity of ccRCC is different [36], especially the higher proportion of low grade in small ccRCC, which contains characteristic higher permeability vascular structure, larger extracellular space and higher microvessel density (MVD) compared with AML.wovf [26, 37].

### Prediction models

This study shows that demographic data and morphological features are also valuable in differential diagnosis, especially gender was identified as the main factor in all prediction models. AML.wovf is significantly more common in females [15], cystic degeneration and pseudocapsule are rare, and angular interface reported previously is common [14, 25], all of which are helpful for differentiation [38]. Our study also shows that enhanced scanning phase is more valuable than PCP in differential diagnosis, especially CMP. The enhancement degree determined by ROI (2) and the heterogeneous degree determined by ROI (3) were identified as the main factors in prediction models of each enhanced phase, indicating the importance of the combination with these two quantitative parameters reflecting the degree of enhancement and homogeneity in differential diagnosis. Among them, the prediction model in CMP has the best performance after combining these two quantitative parameters as well as demographic data and morphological features (AUC = 0.986 [95% CI: 0.970–1.000], accuracy = 93.3%, sensitivity = 95.9%, specificity = 87.1%), which has excellent diagnostic value. We also found that even if the subjective morphological feature (cystic degeneration) is removed from the prediction model in CMP, the diagnostic performance of the model that only depends on objective features (quantitative data of CMP [RER_CMP (2) and SHR_CMP] combined with demographic data) is also very good (AUC = 0.973 [95% CI: 0.948–0.997], accuracy = 91.4%, sensitivity = 95.9%, specificity = 80.6%), showing the potential of quantitative analysis based on single CMP in differentiating AML.wovf from ccRCC.

## Limitations

Our study had several limitations. First, this study is a retrospective study of a small sample from a single center, and the selection of samples may be biased. The placement of ROI in most enhancing area is also subjective as it will depend on the observer's assessment and can be variable. Therefore, the results of our study need to be verified by a large sample from multi-centers. Second, the histopathological basis reflected by quantitative data in this study needs to be further verified by comparative study between imaging and pathology. Third, the difference of scanning phase and contrast agent will affect the value of enhanced quantitative parameters, especially HDT. Although we have applied a relative ratio with HDP to weaken this effect, its impact on final diagnostic performance needs further study. Fourth, we have not performed texture analysis on these samples and further compared with the results of our study, the differences and correlations between these two quantitative analysis methods need to be explored.

## Conclusion

In conclusion, the specific ROI combination that reflects the differences of enhancement degree and heterogeneous degree can be used as a simply convenient quantitative tool to differentiate AML.wovf from ccRCC, among which single CMP has the highest diagnostic performance and great application potential.

## Data Availability

Not applicable.

## Abbreviations

AML: Angiomyolipoma
AML.wovf: AML without visible fat
AUC: Area under the curve
AVT: Attenuation value of tumor
ccRCC: Clear cell renal cell carcinoma
CMP: Corticomedullary phase
EP: Excretory phase
HDT: Heterogeneous degree of tumor
NEV: Net enhancement value
NP: Nephrographic phase
PCP: Pre-contrast phase
RER: Relative enhancement ratio
ROC: Receiver operating characteristic
ROI: Region of interest
SHR: Standardized heterogeneous ratio
